# Waveguide-Integrated Light-Emitting Metal–Insulator–Graphene Tunnel Junctions

**DOI:** 10.1021/acs.nanolett.2c04975

**Published:** 2023-04-25

**Authors:** Lufang Liu, Alexey V. Krasavin, Jialin Li, Linjun Li, Liu Yang, Xin Guo, Daoxin Dai, Anatoly V. Zayats, Limin Tong, Pan Wang

**Affiliations:** † State Key Laboratory of Modern Optical Instrumentation, College of Optical Science and Engineering, Zhejiang University, Hangzhou 310027, China; ‡ Department of Physics and London Centre for Nanotechnology, King’s College London, Strand, London WC2R 2LS, U.K.; § Jiaxing Key Laboratory of Photonic Sensing & Intelligent Imaging, Jiaxing 314000, China; ∥ Intelligent Optics & Photonics Research Center, Jiaxing Research Institute Zhejiang University, Jiaxing 314000, China

**Keywords:** tunnel junction, metal−insulator−graphene, light-emitting, waveguide, plasmonics

## Abstract

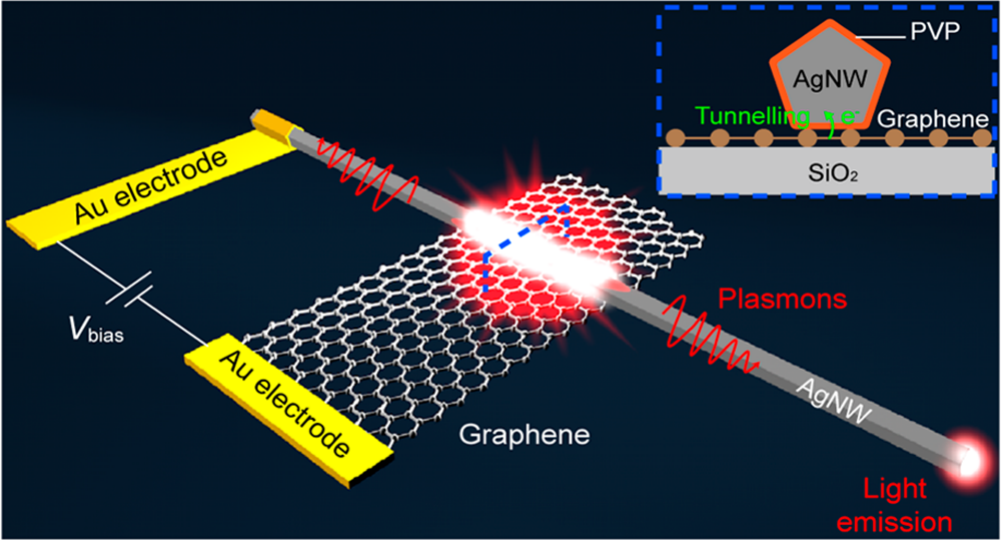

Ultrafast interfacing of electrical and optical signals at the nanoscale is highly desired for on-chip applications including optical interconnects and data processing devices. Here, we report electrically driven nanoscale optical sources based on metal–insulator–graphene tunnel junctions (MIG-TJs), featuring waveguided output with broadband spectral characteristics. Electrically driven inelastic tunneling in a MIG-TJ, realized by integrating a silver nanowire with graphene, provides broadband excitation of plasmonic modes in the junction with propagation lengths of several micrometers (∼10 times larger than that for metal–insulator–metal junctions), which therefore propagate toward the junction edge with low loss and couple to the nanowire waveguide with an efficiency of ∼70% (∼1000 times higher than that for metal–insulator–metal junctions). Alternatively, lateral coupling of the MIG-TJ to a semiconductor nanowire provides a platform for efficient outcoupling of electrically driven plasmonic signals to low-loss photonic waveguides, showing potential for applications at various integration levels.

Quantum-mechanical tunneling enables the transport of electrons across a nanoscale gap between two conducting electrodes, via either elastic or inelastic mechanisms and at a time scale of few femtoseconds.^1,2^ For the elastic electron tunneling process, electrons tunnel across the barrier layer without energy loss, emerging as hot electrons in the receiving electrode, while for the inelastic electron tunneling (IET) process, electrons lose part of their energy by exciting electromagnetic modes of the tunnel junction^3−6^ or generating excited electronic and vibrational states of molecules/atoms in the gap.^7−11^ Since the first discovery in a planar metal–insulator–metal tunnel junction (MIM-TJ),^3^ direct electrical excitation of optical modes by IET has attracted extensive research interest^12−25^ due to its potential to create electrically driven optical sources with an ultrahigh modulation bandwidth (THz level), an ultrasmall footprint (nanometer scale), and a low operation voltage (several volts), which are highly required for high-speed integrated optoelectronic circuits.

The external quantum efficiency (EQE) of an IET-based optical source is defined by the ratio between the number of outcoupled photons (or plasmons) to the total number of tunnelled electrons. It is determined not only by the IET efficiency (the ratio between the inelastic tunneling rate related to the excitation of electromagnetic modes in the junction and the total electron tunneling rate) but also by the loss of these modes in the junction region and their outcoupling efficiency to the desired output, such as free-space light and waveguided plasmonic or photonic modes.^4,5^ Recently, by combining tunnel junctions with optical nanoantennas, it was shown that the efficiency of generation of free-space photons can be greatly enhanced via the large local density of optical states (LDOS) in the tunnel junctions (which greatly increases the IET efficiency) and the high far-field radiation efficiency of the optical nanoantennas,^13,14,17−19,23,24,26^ with the EQE of light emission reaching the levels up to ∼2%.^17^ However, it remains a great challenge to couple the metal–insulator–metal (MIM) plasmonic modes excited in the nanoscale tunneling gap between the two electrodes to technologically appealing modes of a waveguide^12,21,27−30^ as opposed to omnidirectional emission into free-space light. The two main factors that limit the EQE are the high propagation loss of the highly confined MIM plasmonic modes and the dramatic momentum and modal size mismatch between the highly confined MIM plasmonic modes and the plasmonic or photonic waveguided modes,^4,21,31,32^ which greatly limit the outcoupling of the excited MIM plasmonic signal to the optical circuits. In particular, the extremely short propagation length (few hundreds of nanometers^4,21^) of the MIM plasmonic modes greatly limits the contribution of the plasmonic signal generated far from the edge of the tunnel junction region when its lateral size is significantly larger than the propagation length. Additionally, the dramatic momentum and modal size mismatch further makes it difficult for the MIM mode energy reaching the edge of the MIM-TJ to be outcoupled into the optical circuits. These issues can be alleviated by using nanoantenna designs^15,30^ or local excitation approaches based on scanning tunneling microscope tips,^12^ having a small lateral size to achieve high outcoupling efficiency of the MIM modes into the waveguided modes, but the overall waveguided output optical power (important for on-chip applications) in this case is quite limited due to the intrinsically low input electrical power of such tunnel junctions. Recently, it was demonstrated that the outcoupling of the highly confined MIM mode into the extended waveguided modes can be improved by decreasing the electrode thickness and increasing the interface roughness of the MIM-TJs.^16,21,33,34^

Here, we break through the limitation of low-efficiency waveguided output of inelastic tunneling-based light sources by developing waveguide-integrated metal–insulator–graphene tunnel junctions (MIG-TJs) based on an organic layer-coated silver nanowire (AgNW) interfaced with graphene. The use of graphene instead of one of the metal electrodes of conventional tunnel junctions not only eliminates the highly lossy MIM modes and therefore ensures efficient delivery of the IET-excited optical signal to the edge of the tunnel junction where it can be extracted but also offers a small mode mismatch between the tunnel junction region and the plasmonic nanowire waveguide, which enables efficient coupling (∼70%) of the optical signal from the edge of the junction to the output AgNW waveguide. Furthermore, we demonstrate the coupling of the AgNW-integrated tunnel junction with a low-loss semiconductor nanowire waveguide, thus realizing efficient outcoupling of the IET-excited optical signal to both highly integrated plasmonic and low-loss photonic channels. Finally, as a proof of principle, a direct electrical modulation of the outputted optical signal has been also demonstrated.

Figure 1A shows a schematic illustration of a plasmonic waveguide-integrated MIG tunnel structure, produced by the intersection of an AgNW with a graphene monolayer. A MIG tunnel barrier in this case is formed by a layer of polyvinylpyrrolidone (PVP) naturally covering the AgNW surface (Figure 1A, inset). The AgNW does not only act as one of the electrodes of the tunnel junction but also as a plasmonic waveguide seamlessly connected with the tunnel junction. When a bias is applied between the AgNW and the graphene, electrons tunnel across the nanometer-scale molecular gap from occupied states in graphene to unoccupied states in Ag (Figure 1B). Part of the electrons tunnel inelastically losing their energy to excite plasmonic modes in the junction region. These modes propagate across the edges of the tunnel junction into the output AgNW with high efficiency due to the good mode matching between the tunnel junction region and the AgNW waveguide. Finally, the propagating plasmonic signal is converted to photons at the tip of the AgNW via scattering (Figure 1A).

**Figure 1:**
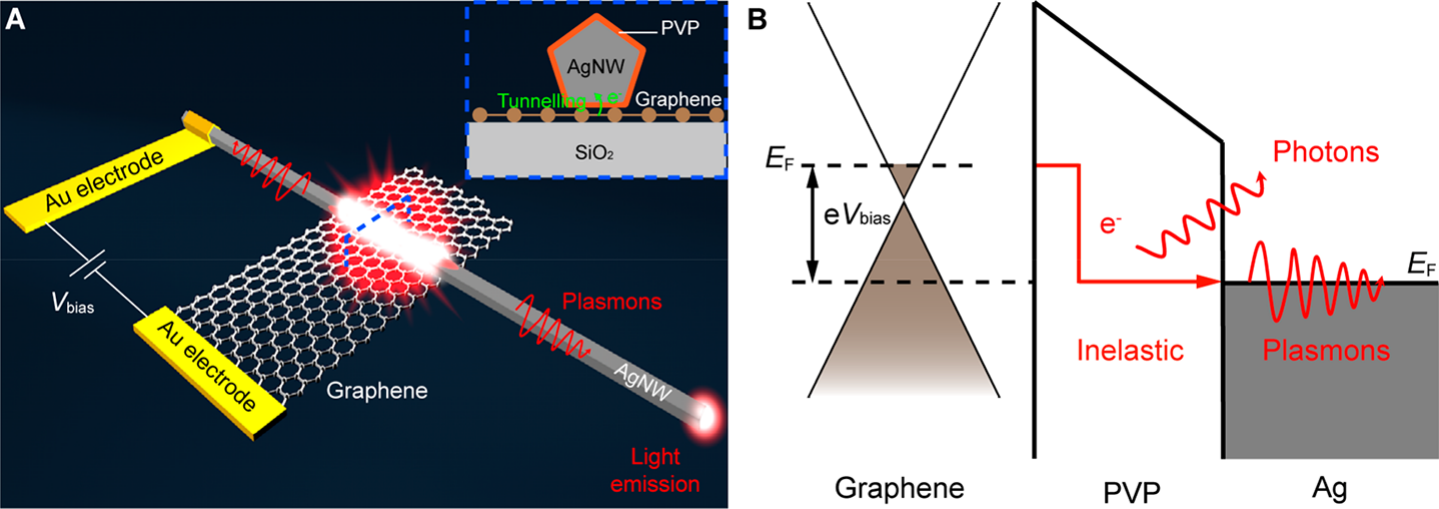
Design of an AgNW-integrated MIG-TJ. (A) Schematic representation of an AgNW-integrated MIG-TJ, which consists of an AgNW on the top of a graphene monolayer separated by a dielectric spacer (PVP). Inset: cross-sectional view of the MIG-TJ (taken at the position marked by a blue dashed box in the main scheme). (B) Energy diagram of the MIG-TJ under the application of a bias voltage of *V*_bias_.

In order to further understand the characteristics of the MIG-TJs and their difference with conventional MIM-TJs, fully vectorial 3D finite element method numerical simulations were performed (Supporting Information Section 1). A point electric dipole directed along the *z*-axis, modeling the tunneling current,^4,5,13,18,35^ was placed at regularly spaced positions along the junction length with a 10 nm interval to mimic the tunneling events (Figure 2A,B). The junction length was set to be 1 μm to study the most competitive scenario for the MIM-TJ and MIG-TJ. For the fair comparison of the designs, a uniform thickness of the tunnel barrier (the thickness of the PVP layer was set to be 2.5 nm) along the nanowire was considered. The probability of the excitation of the propagating plasmonic modes by the tunneling electrons in the junction region is proportional to the LDOS associated with these modes.^4,5,18^ Therefore, the mode LDOS (ρ_mode_) for the MIM-TJ and MIG-TJ was first estimated from ρ_mode_ = ρ_0_ × (*P*_mode_/*P*_0_), where ρ_0_ is the vacuum LDOS, ρ_mode_ is the corresponding mode power excited by the point electric dipole, and *P*_0_ is the radiated power of a dipole of an equal dipole moment in a vacuum environment.^4,36^ The normalized mode LDOS (ρ_mode_/ρ_0_) of the MIG-TJ is about 2 orders of magnitude lower than that for the MIM-TJ (Supporting Information Section 2). Figure 2C presents the calculated optical power of the output waveguided modes in the AgNW as a function of the dipole position for 800 nm emission wavelength. When the dipole position is very close to the edge of the junction, the optical power of the output waveguided modes in the AgNW for the MIM-TJ is about 10 times higher than that for the MIG-TJ. However, with the further increase of the dipole position, the optical power of the output waveguided modes in the AgNW for the MIM-TJs dramatically decreases compared with that for the MIG-TJs. As an example, the optical power of the output waveguided modes in the AgNW for the MIM-TJ is about 15 times lower than that for the MIG-TJ at the dipole position of *d* = 500 nm. These differences can be also seen from the corresponding cross-sectional maps of the normalized *z*-component of the electric field (*E*_*z*_) (Figure 2D–G). As expected, for the MIM-TJ (Figure 2D,F), a highly confined MIM plasmonic mode is excited and subsequently propagates inside the tunneling gap to its edge with a very short propagating length of ∼0.3 μm. Therefore, only a small fraction of the total mode energy excited by the tunneling sources in the MIM-TJ can reach the edge of the junction. Furthermore, due to the dramatic mode mismatch between the highly confined MIM plasmonic mode and the propagating plasmonic mode in the output nanowire waveguide (cf. Figure 2H,I), it is extremely difficult for the MIM plasmonic mode reaching the junction edge to be coupled to the nanowire plasmonic mode, as the coupling coefficient is proportional to the corresponding mode overlap.^37^ The efficiency of this process was estimated to be as low as ∼10^–3^. By contrast, for the MIG-TJ (Figure 2E,G), plasmonic mode of the nanowire–graphene structure having a much higher propagation length (∼6.5 μm) can be directly excited by the dipolar tunneling source.^19,38^ Furthermore, the modes reaching the junction edge can couple to the mode of the plasmonic nanowire with an efficiency of ∼0.7 benefiting from a small mode mismatch between the modes (cf. Figure 2J,K). For the values of propagation length and coupling efficiency at other wavelengths see Table S1. These advantages are so prominent that they prevail over the higher initial excitation efficiency of the MIM-TJ mode related to the LDOS when the dipole position is larger than ∼200 nm from the junction edge (Figure 2C), giving the MIG-TJ an overall superiority in the total waveguided output power when the junction length is larger than a few propagation lengths of the MIM mode (see the Supporting Information Section 3 for calculated results for junction lengths of 0.5 and 0.1 μm). Particularly, the total power in the output waveguide generated by the 1 μm MIG-TJ is about 1.3, 1.5, and 2.5 times larger than corresponding values for 1, 0.5, and 0.1 μm MIM-TJs, respectively (see output power magnitudes in the Supporting Information Section 3). Moreover, the total waveguided output power of the MIG-TJ can be further increased by increasing its length due to the long propagation length of the plasmonic mode of the nanowire–graphene structure. If miniaturization is the priority, then MIM-TJ designs with ∼0.1 μm sizes may offer better performance. It is worth noting that for MIM-TJs with a micrometer-scale junction length, the outcoupling of the highly confined MIM mode energy to the extended waveguided modes can be improved by decreasing the electrode thickness and increasing the interface roughness of the MIM-TJs,^16,21,33,34^ thus obtaining a higher overall waveguided optical power.

**Figure 2:**
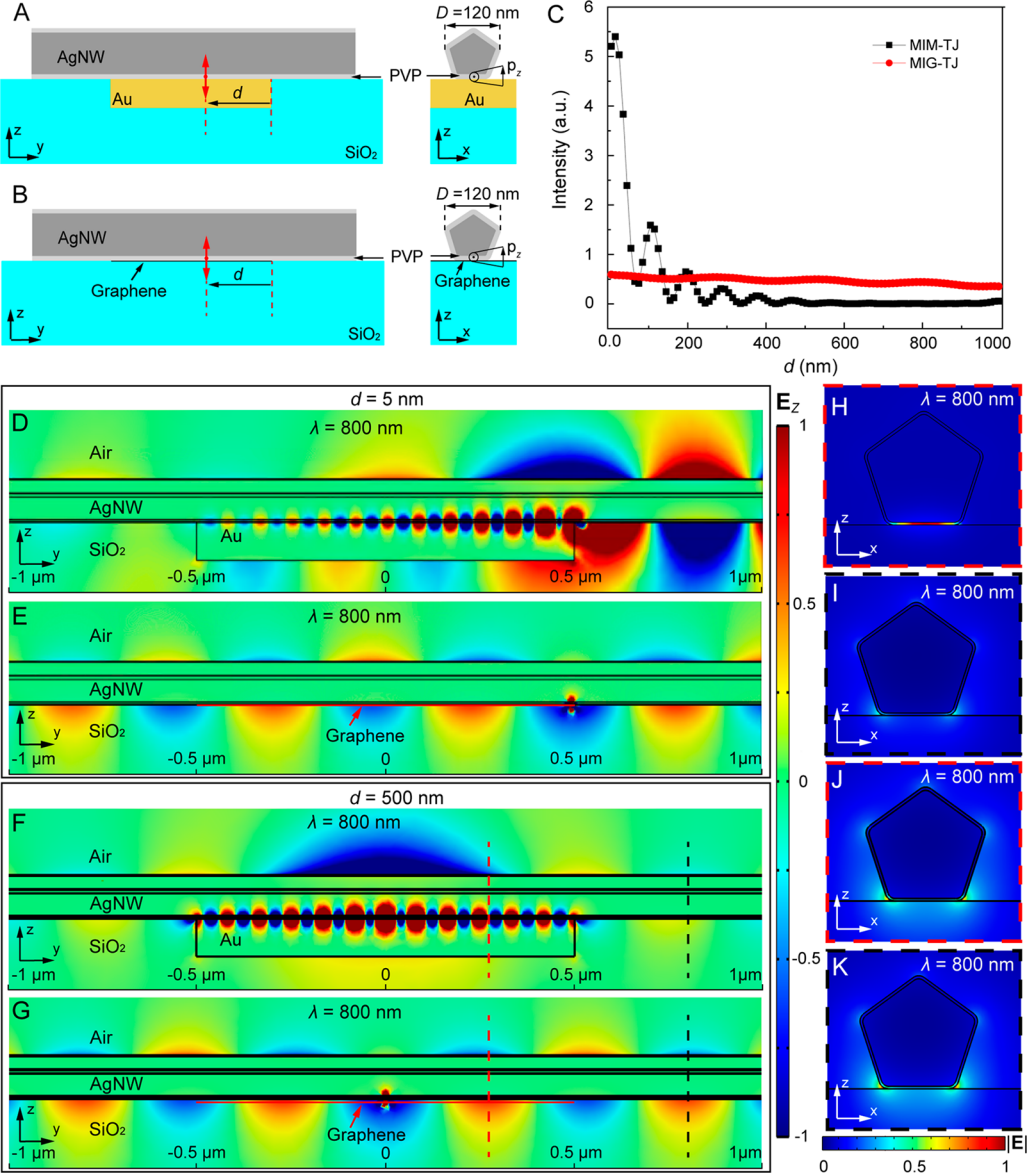
Numerical simulations of MIM-TJ and MIG-TJ. (A, B) Schematic illustrations of the MIM-TJ and MIG-TJ used for simulations. (C) Calculated optical power in the output AgNW (integrated at the right end of the AgNW) as a function of the dipole position for 800 nm emission wavelength. (D–G) Cross-sectional maps of normalized *z*-component of the electric field *E*_*z*_ excited by a *z*-oriented point electric dipole placed at a distance of 5 nm from the right edge of the tunnel junction (D, E) and in its center (F,G) for MIM-TJ (D, F) and MIG-TJ (E, G). The length and thickness of the Au electrode are 1 μm and 80 nm, respectively. (H, I) Normalized intensity |*E*| distributions of the plasmonic modes in the AgNW-integrated MIM-TJ taken at the planes perpendicular to the waveguide marked by red and black dashed lines in (F). (J, K) Normalized intensity |*E*| distributions of the plasmonic modes in the AgNW-integrated MIG-TJ taken at the planes perpendicular to the waveguide marked by red and black dashed lines in (G).

Figure 3A shows an optical microscopy image of an AgNW-integrated MIG tunneling device, which was fabricated as follows (Supporting Information Section 4): a graphene monolayer (Figure S2A) was first transferred onto a silica-coated silicon wafer, with its right part connected electrically to a gold electrode. Then, an AgNW, which has a pentagonal cross section and an outer diameter of ∼120 nm (Figure 3B), was transferred onto the substrate by micromanipulation,^39^ with its right part partially crossing the graphene monolayer and the left end connected electrically to another gold electrode. The PVP molecules covering the AgNW produce an insulating layer with an average thickness of ∼2.5 nm (Figure S2B). The total junction length was about 4 μm, and the right end of the AgNW was about 3 μm away from the junction region (Figure 3B). Figure 3C presents a typical current–voltage curve for the tunneling device (black dots), showing the superlinear dependence characteristic to the tunneling at low biases. The red line shows a fit of the experimental data with a theoretical curve calculated using the Simmons model^40^ (barrier height 3.1 eV, gap size 2.65 nm), which demonstrates an excellent agreement with the experimental results.

**Figure 3:**
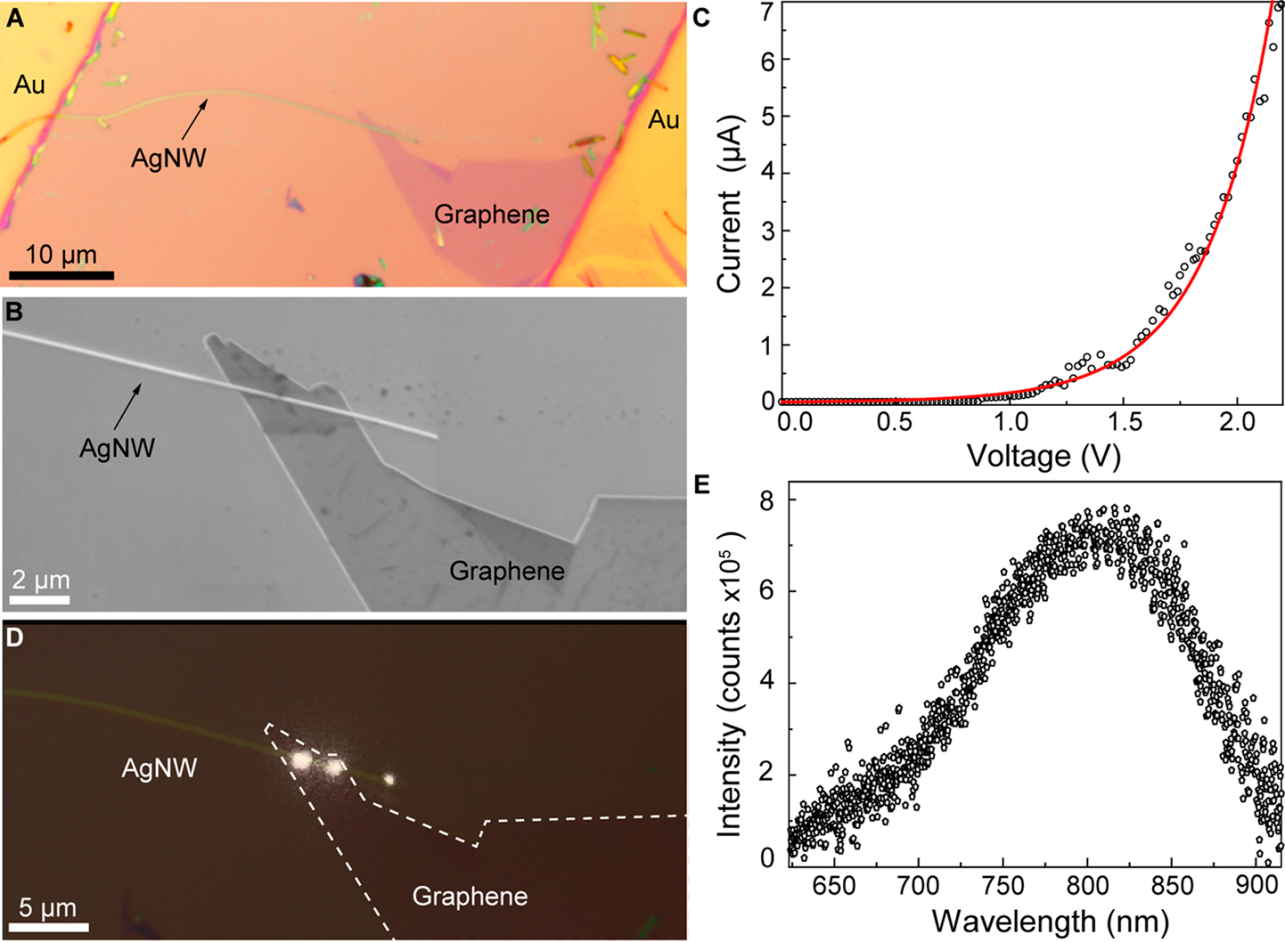
Electrical and optical properties of an AgNW-integrated MIG-TJ. (A) Optical micrograph of an AgNW-integrated MIG-TJ. (B) Enlarged SEM image of the MIG-TJ. (C) Current–voltage characteristic of the tunneling device. The black open circles present the experimentally measured results, while the red line corresponds to a theoretical fit obtained using the Simmons model. (D) Detected emission from the tunneling device (*V*_bias_ = 2.2 V) superimposed with its bright-field optical micrograph. The white dashed line shows the outline of the graphene layer. (E) Emission spectrum of the tunneling device collected from the nanowire tip at *V*_bias_ = 2.2 V.

With a bias of 2.2 V applied between the AgNW and the graphene, clearly visible light emission was observed from the device (Figure 3D; see the Supporting Information Section 5 for the optical characterization setup), both from the MIG-TJ region and the nanowire tip. After the waveguiding loss in the AgNW (about 0.87 dB/μm for a 130 nm diameter AgNW^41^) was deducted, the coupling efficiency from the total optical energy generated by the IET process in the MIG-TJ to the mode of the AgNW in both directions is estimated to be ∼36%. Importantly, in the case of an AgNW-integrated MIM tunneling device, it was difficult to observe light emission at the nanowire tip (it can only be observed near the junction region^32^) due to the high loss of the excited MIM plasmonic mode and the dramatic mode mismatch between the MIM plasmonic mode and the propagating plasmonic mode in the nanowire (Figure 2D,E), resulting in their low coupling efficiency. Oppositely, due to a lower carrier concentration, the use of graphene instead of one of the metal electrodes of conventional tunnel junctions not only minimizes the junction absorption losses, boosting the delivery of the optical signal from the entire junction region to the junction edge, but also only slightly perturbs the nanowire plasmonic mode, leading to a very good mode coupling between the tunneling region and the output waveguide (Figure 2F,G). This leads to an excellent waveguided output performance of the AgNW-integrated MIG-TJ light source. The uneven light emission from the junction region directly into the far-field can be attributed to a perturbation due to a nonuniform thickness of the tunnel barrier along the wire, mainly caused by the nonuniform thickness of the PVP molecules on the AgNW surface (Figure S2B) and residual polymers on the graphene surface after the transfer process. This can be improved by substituting PVP molecules on the AgNW surface with a uniform layer of alternative materials (e.g., self-assembled alkanethiols^32,42^) and optimizing the cleaning step after the graphene transfer.

Figure 3E presents an emission spectrum of the tunneling device at *V*_bias_ = 2.2 V measured from the nanowire tip. The emission spectrum is essentially broadband, which is fundamentally related to the nature of the IET process, producing photonic and/or plasmonic excitations of all energies below *eV*_bias_.^3,4,19,38^ From the measured emission power at the nanowire tip regions together with the tunneling current of ∼7 μA at 2.2 V, the EQE for the waveguided plasmonic output channel in both directions was estimated to be 2 × 10^–6^ (Supporting Information Section 6). This value is about 10 times lower than that of tunneling devices with waveguided output based on nanoantenna designs^15,30^ or scanning tunneling microscope tips^12^ (tunneling current ∼10 nA), which is mainly due to the lower LDOS of the MIG-TJs. At the same time, the MIG-TJs demonstrated here can provide a much higher overall waveguided optical power important for practical applications because of their higher electrical input power provided by the longer junction length.

High propagation loss in the plasmonic waveguide prevents the long-distance transmission of optical signals, limiting the application areas of the light-emitting MIG-TJs. Therefore, in addition to coupling to plasmonic waveguides with strong optical confinement, it is highly desired to integrate the light-emitting MIG-TJs to low-loss photonic waveguides,^27−29^ which is attractive for the incorporation of electrically driven light-emitting devices into low-loss dielectric circuits. To realize this, we coupled an AgNW-integrated MIG-TJ to a low-loss semiconductor nanowire waveguide at the junction area via the optical near-field interaction and demonstrated an electrically driven tunneling device with both plasmonic and photonic outputs (Figure 4A).

**Figure 4:**
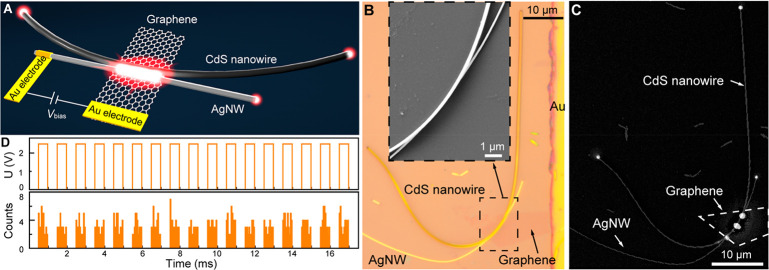
Coupling an AgNW-integrated MIG-TJ to a low-loss photonic waveguide and electrical modulation of the output optical signal. (A) Schematic illustration of an electrically driven tunneling device having both plasmonic and photonic output channels, which is based on an AgNW-integrated MIG-TJ coupled with a CdS nanowire waveguide. (B) Optical micrograph of an as-fabricated tunneling device. Inset: SEM image of the device showing the junction region. (C) Detected emission from the tunneling device (*V*_bias_ = 2.5 V) superimposed with a SEM image of the device. The white dashed line shows the outline of the graphene layer. (D) Top: time dependence of the bias applied to the device (square waveform switched between 0 and 2.5 V at a frequency of 1 kHz). Bottom: the corresponding output optical signal detected from the end of the CdS nanowire.

Experimentally, an AgNW-integrated MIG-TJ was first constructed using the approach described above. Then, a CdS nanowire^43^ with a propagation loss less than 1 dB/mm^44^ was brought into a side-by-side contact with the MIG-TJ by micromanipulation (Supporting Information Section 7), forming an electrically driven tunneling device with both plasmonic and photonic outputs. Figure 4B presents an optical microscopy image of an as-fabricated tunneling device, in which a CdS nanowire (235 nm in diameter) is coupled with an AgNW-integrated MIG-TJ at the junction region (Figure 4B, inset). The total junction length was about 7 μm, and the free tip of the AgNW having a diameter of 130 nm was about 10 μm away from the junction edge. Upon an application of a bias voltage of 2.2 V to the device, in addition to light emission from the MIG-TJ region and the free tip of the AgNW, two clearly visible light spots could be observed at the ends of the CdS nanowire about 50 μm away from the junction region (Figure 4C), indicating the efficient coupling of the optical signal excited at the tunnel junction into the photonic waveguide and the low-loss propagation of the optical signal in it. Similarly, from the measured emission power at plasmonic and photonic outputs and the tunneling current (8 μA at 2.2 V), the EQEs for the plasmonic and photonic output channels in this device are estimated to be ∼1.8 × 10^–6^ and 1.1 × 10^–6^ (Supporting Information Section 6), respectively. The output ratio between the photonic and plasmonic channels can be further adjusted by tuning the coupling strength and interaction length between the CdS nanowire and the MIG-TJ,^45^ making it possible to couple the electrically driven optical signal from the tunnel junction into the low-loss photonic channel with much higher efficiency.^45,46^

One of the major advantages of electrically driven plasmonic tunnel junctions is their potentially ultrahigh operation bandwidth and therefore ultrafast data encoding capability. Fundamentally, the response time of plasmonic tunnel junctions is only limited by the tunneling time of the electrons, which is typically on a femtosecond scale.^1,2^ Practically, the response time of plasmonic tunnel junctions is determined by a slower RC time of the device, which, however, can be efficiently reduced to picoseconds or even subpicoseconds time scales (terahertz operation bandwidth) by the miniaturization. Therefore, the AgNW-integrated MIG-TJ devices developed here, featuring nanoscale dimensions, present a very promising platform offering a chance to realize the full potential of ultrahigh-bandwidth operation. Here, as a proof of principle, the possibility of direct electrical modulation of the waveguided output signal was demonstrated in the case of integration with the photonic waveguides based on the CdS nanowires. Instead of a constant bias, a bias with a square voltage waveform was applied to the tunneling device, and a modulated light signal emitted from the end of the CdS nanowire was detected with a single-photon detector (Supporting Information Section 8). Following the alternative switch of the bias between 0 and 2.5 V at 1 kHz frequency (Figure 4D, top panel), the optical output from the CdS waveguide shows pronounced modulation in the emission intensity (Figure 4D, bottom panel), with a modulation depth reaching almost 100%. The relatively low modulation frequency demonstrated here is mainly restricted by the detection limit of the single-photon detector, as an increase in the frequency of the driving bias causes a decrease in photon counts in each pulse. Thus, it does not correspond to a device-related constraint, and in principle the direct electrical modulation frequency of the tunneling device can reach a value of ∼0.3 THz (estimated on the basis of the structural parameters of the tunneling junction), which is only limited by the RC time constant of the device.^14,16^

In conclusion, we have experimentally demonstrated nanoscale waveguide-integrated light-emitting MIG-TJs, featuring waveguided output with broadband spectral characteristics. Taking advantage of efficient outcoupling of IET-excited optical modes in the tunnel junctions not only into highly confined plasmonic waveguides but also to low-loss photonic waveguides, the developed technological platform provides low-loss integration of light-emitting tunnel junctions with plasmonic or photonic devices and circuits at various levels of integration. Because of the nature of the tunneling process, the emission spectrum of the device is fundamentally broadband, which furthermore can be controlled by the applied bias, offering a valuable versatility for applications. Additionally, the spectrum of the waveguided signal can be tuned by fabricating waveguide-based plasmonic Fabry–Perot cavities^47^ or integrating MIG-TJs with their photonic ones. The plasmonic signal can be further launched unidirectionally by fabricating an aperiodic groove array reflector on one side of the waveguide.^48^ Overall, waveguide-integrated light-emitting MIG-TJs provide a very promising technological platform for the implementation of ultracompact and ultrafast electrically driven light sources for integrated photonics.

## Supporting Information Available

Numerical simulations; calculated results for the MIM-TJ and MIG-TJ; calculated dependence of output optical power on position of tunneling source; fabrication of tunneling devices; optical characterization setup; estimation of external quantum efficiency (EQE); micromanipulation process of coupling a CdS nanowire with a MIG-TJ; setup for direct electrical modulation of light-emitting MIG-TJs (PDF)
