# Supplementary material for: Waveguide-Integrated Light-Emitting Metal–Insulator–Graphene Tunnel Junctions
Source: Nano Lett. 2023 Apr 25;23(9):3731–8. doi: 10.1021/acs.nanolett.2c04975 (PMC10176563; doi:10.1021/acs.nanolett.2c04975)
Supplement: Supplementary file 1 — nl2c04975_si_001.pdf [file nl2c04975_si_001.pdf]

Supporting Information for

Waveguide-integrated light-emitting metal-insulator-graphene tunnel junction

Lufang Liu[1], Alexey V. Krasavin[2], Jialin Li[1], Linjun Li[1,3,4], Liu Yang[1], Xin Guo[1,3,4], Daoxin Dai[1], Anatoly Zayats[2,], Limin Tong[1,*], Pan Wang[1,3,4,*]*

[1]State Key Laboratory of Modern Optical Instrumentation, College of Optical Science and Engineering, Zhejiang University, Hangzhou 310027, China

[2]Department of Physics and London Centre for Nanotechnology, King's College London, Strand, London WC2R 2LS, UK

[3]Jiaxing Key Laboratory of Photonic Sensing & Intelligent Imaging, Jiaxing 314000, China

[4]Intelligent Optics & Photonics Research Center, Jiaxing Research Institute Zhejiang University, Jiaxing 314000, China

This PDF file includes:

Sections 1 to 8

Table S1, S2

Figures S1 to S5

References

Section 1. Numerical simulations

The simulations were performed using a finite element method (COMSOL Multiphysics software). The cross-sectional shape of the AgNW was set to be pentagonal with an outer diameter of 120 nm to match the geometry of its experimental counterparts. The AgNW was covered with a PVP layer with a thickness of 2.5 nm, defining the distance between the bottom surface of the nanowire and the metallic or graphene electrode lying on the substrate. The nanostructure was excited with a point electric dipole directed along the z axis and having a spectral profile $C(\omega) \propto \left(1 - \hbar\omega/eV_{\text{bias}}\right)$, modelling the tunnelling current (here V_{bias} is the applied bias and e is the electron charge)[1,2], for the calculation of the LDOS a unit dipole was used according to the standard procedure[3,4]. To avoid back-reflection of the optical signal, the simulation domain was surrounded by PML layers. The refractive indices of Au, Ag, and PVP are obtained from Refs. 5-7. The optical conductivity of the graphene monolayer was first calculated with an effective medium theory considering the layer thickness of $t = 1$ nm [Ref. 8] and using an analytical theory from Ref. 9. Then, it was converted into a surface conductivity of the implemented infinitely thin graphene layer.

Section 2. Calculated parameters of MIM-TJs and MIG-TJs

Table S1. Comparison of LDOS associated with the tunnelling-excited waveguided mode ($\text{LDOS}_{\text{mode}}$), propagation lengths ($L_{\text{prop}}$) and outcoupling coefficients κ for the MIM-TJ and MIG-TJ. The mode LDOS was estimated from the ratio of the power flow of the mode and the power flow of the equivalent point electric dipole in a vacuum environment[10]. L_{prop} was calculated from 2D eigenmode simulations of the waveguides. The outcoupling coefficients κ were calculated by integrating power flows over a nanoscale region encircling the waveguide surface before and after the junction, taking into account the reflection at the edge of the junction (the refection coefficient was calculated fitting $E_z E_z^*$ profiles in the junction region near the edge). The power flows near the waveguide surface were taken in order to separate the power flow

of the waveguide modes from those corresponding to excited photonic modes, the total power of the waveguided modes were reconstructed using the mode power flow profiles found in the 2D eigenmode simulations. In the case of MIM-TJ they could be estimated only down to the order of magnitude due to the extremely low outcoupling power of the mode and therefore essential impact of low-power stray photonic and plasmonic modes in the extended outer region around the waveguide.

Wavelength (nm)	Normalized mode LDOS		Propagation lengths (μm)		Outcoupling coefficients	
	$\mathrm{LDOS}_{\mathrm{mode}}^{\mathrm{MIM}}$	$\mathrm{LDOS}_{\mathrm{mode}}^{\sigma}$	$L_{\mathrm{prop}}^{\mathrm{MIM}}$	$L_{\mathrm{prop}}^{\sigma}$	κ_{MIM}	κ_{g}
700	2.9×10^3	48	0.19	3.77	$\sim 10^{-3}$	0.69
750	6.1×10^3	48	0.24	4.87	$\sim 10^{-3}$	0.71
800	8.8×10^3	58	0.30	6.52	$\sim 10^{-3}$	0.70
850	8.8×10^3	67	0.35	8.09	$\sim 10^{-3}$	0.68
900	9.3×10^3	71	0.38	9.02	$\sim 10^{-3}$	0.66
950	1.0×10^4	80	0.39	9.61	$\sim 10^{-3}$	0.64
1000	1.4×10^4	99	0.41	16.49	$\sim 10^{-3}$	0.64

Section 3. Calculated dependence of output optical power on position of tunnelling source

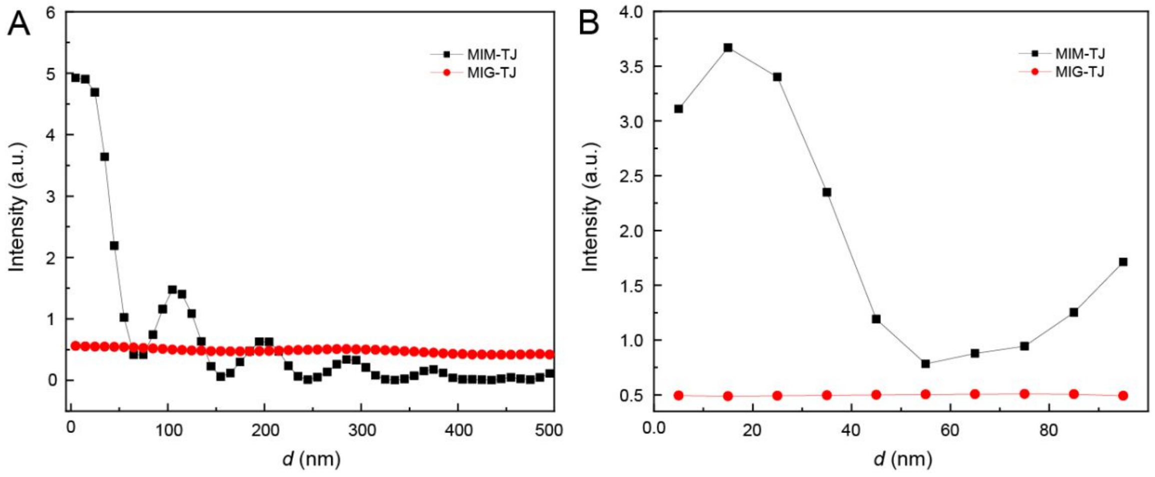

Figure S1. Calculated optical power in the output AgNW as a function of the position of the tunnelling source (point electric dipole) for tunnel junctions with the lengths of (**A**) 0.5 and (**B**) 0.1 μm for 800 nm emission wavelength.

Table S2. Normalized total powers in the output waveguide generated by tunnel junctions with different lengths.

	Length of junction		
	1 μm	0.5 μm	0.1 μm
Output total power for MIM-TJ (a.u.)	37	34	19
Output total power for MIG-TJ (a.u.)	47	31	5

Section 4. Fabrication of tunnelling devices

MIG-TJs were fabricated on a silicon wafer covered with a 300-nm-thick SiO_2 layer. The Au electrodes were fabricated by first defining the patterns using direct laser writing (HEIDELBERG DWL66+), and then depositing 5-nm-thick Cr and 60-nm-thick Au films using magnetron sputtering (DISCOVERY-635) under a direct-current power of 100 W and 200 W, respectively. The lift-off process was performed in acetone.

Graphene monolayers were mechanically exfoliated from a bulk carbon crystal onto a silicon wafer using an adhesive tape[11], the number of monolayers in a graphene flake can be determined by Raman spectroscopy[12] . Figure S2**A** shows a Raman spectrum (measured in the range of 1250–3000 cm^{-1} at room temperature) of an exfoliated graphene flake shown in Figure 3. Two characteristic peaks can be observed at 1586 and 2676 cm^{-1}, and the intensity of the G' peak is higher than that of the G peak, which confirms that the graphene flake is monolayer.

AgNWs with an average outer diameter of 120 nm were obtained from XFNANO (XFJ26 7440-22-4) and drop-casted on a glass slide. To fabricate waveguide-integrated MIG tunnelling devices, a graphene monolayer was first picked up by a piece of poly(methyl methacrylate) (PMMA) film and transferred onto the silicon substrate to make a contact with one of the Au electrodes, with the process controlled upon observation in an optical microscope. Afterwards, the PMMA film was removed by sequentially washing the structure with chloroform and isopropyl alcohol. Finally, an AgNW was picked up from the glass slide with a fiber taper[13], and transferred onto the silicon substrate with one of its end partially overlapping the

graphene monolayer to form a MIG-TJ and the other end connected electrically to another Au electrode.

The thickness of PVP molecules covering the surface of the AgNWs was determined from TEM images (Hitachi HT7700, operated at 100 kV). The AgNW samples used for TEM imaging were prepared by transferring single AgNWs onto a TEM grid with a fiber taper[13]. As shown in Figure S2**B**, a native organic layer of PVP with an average thickness of ~2.5 nm existing on the surface of the AgNW can be easily observed.

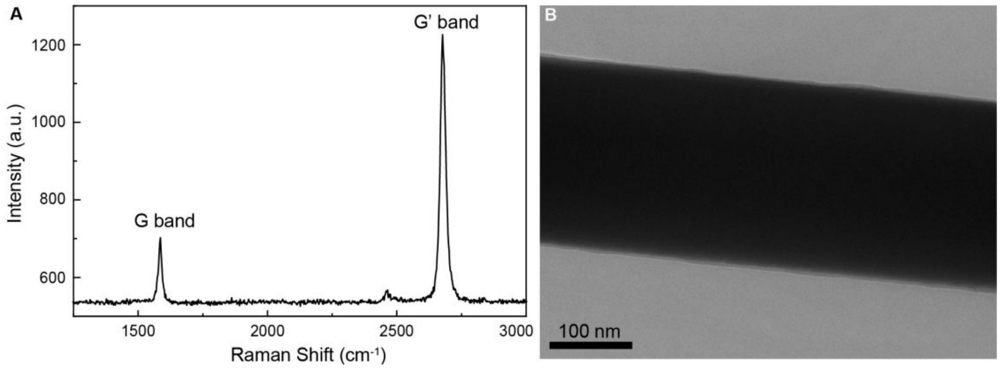

Figure S2. (**A**) Raman spectrum of the graphene flake shown in Figure 2. (**B**) TEM image of the edge of an AgNW showing a native organic layer of PVP covering the AgNW.

Section 5. Optical characterization setup

The optical properties of light-emitting MIG-TJs were characterized by using an optical microscope equipped with a spectrometer (Kymera 193i) and an EMCCD (Andor iXon Ultra), as schematically shown in Figure S3**A**. Briefly, a DC voltage bias from a sourcemeter (Keithley 2611B) was applied to a light-emitting MIG-TJ between the AgNW and the graphene. The light emission from the MIG-TJ was collected by a 100× long working distance objective (NA = 0.9, TU Plan Fluor, Nikon) and directed through a beam splitter to the EMCCD for imaging and to the spectrometer for spectral analysis. The obtained emission spectrum was then corrected by the spectral response of the measurement setup:

$$P(\lambda) = \frac{P_{\text{meas}}(\lambda)}{T(\lambda)},\#(S1)$$

where $P_{\text{meas}}(\lambda)$ is the emission spectrum measured by the spectrometer, $P(\lambda)$ is the corrected emission spectrum, and $T(\lambda)$ is the overall spectral transfer function of the measurement setup, which includes the wavelength-dependent transfer functions of all individual optical elements in the optical path (microscope objective, mirrors, an optical fiber and a grating), detection efficiency and window transmittance of the EMCCD used in the spectrometer. The obtained transfer function is presented in Figure S3**B**.

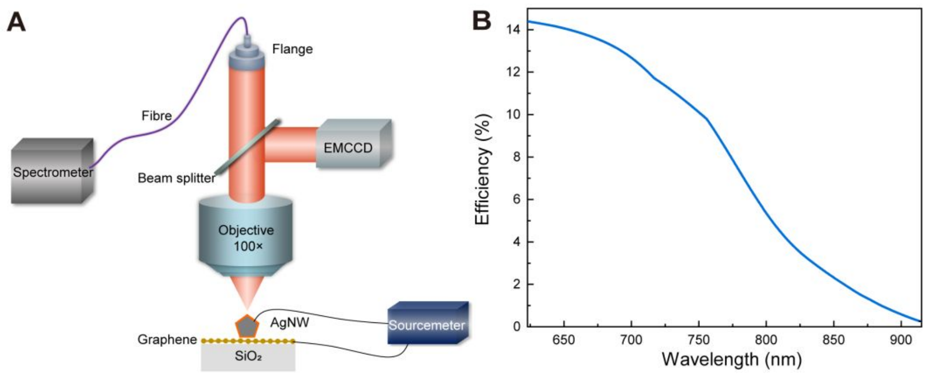

Figure S3. (**A**) Schematic diagram of the optical characterization setup. (**B**) Wavelength-dependent spectral response of the measurement setup used for the collection of the emission spectrum.

Section 6. Estimation of external quantum efficiency (EQE)

For the tunnelling device presented in Figure 3, the EQE of the waveguided plasmonic output channel is defined by the ratio between the number of outcoupled plasmons to the total number of tunnelling electrons in a given period of time:

$$EQE_{\text{WG - plasmon}} = N_{\text{WG - plasmon}}/N_{\text{e}},\#(S2)$$

where, as schematically shown in Figure S4**A**, $N_{\text{WG - plasmon}}$ is the total number of plasmons excited by the MIG-TJ in the AgNW mode in both directions, and N_{e} is the total number of tunnelling electrons. To estimate $N_{\text{WG - plasmon}}$, the total numbers of photons emitted at the AgNW end (N_{AgNW}) per second was first

estimated by integrating the corrected spectral distribution of the overall intensity emitted from the nanowire tip (recovered from the signal collected by the objective with NA = 0.9) and then dividing it by the photon energy (for simplicity assuming that all the emitted photons have the same wavelength of 850 nm). Then N_{AgNW} was corrected for the propagation loss of the AgNW waveguide and finally multiplied by 2 to obtain $N_{\mathrm{WG-plasmon}}$, representing the overall EQE in both directions.

For the tunnelling device presented in Figure 4, as schematically shown in Figure S4**B**, the EQE for waveguided photonic output is further calculated, defined by the ratio between the number of outcoupled photons in waveguided photonic channel to the total number of tunnelling electrons in a given period of time:

$$EQE_{\mathrm{WG-photon}} = N_{\mathrm{WG-photon}}/N_{\mathrm{e}}, \#(S3)$$

where $N_{\mathrm{WG-photon}}$ is the total number of photons excited by the MIG-TJ in the CdS nanowire mode in both directions. To estimate $N_{\mathrm{WG-photon}}$, the total number of photons emitted at the CdS nanowire end ($N_{\mathrm{CdS\ nanowire}}$) was first obtained with the approach introduced above. Then $N_{\mathrm{CdS\ nanowire}}$ was corrected for the propagation loss of the waveguide and finally multiplied by 2 to obtain $N_{\mathrm{WG-photon}}$, representing the overall EQE in both directions**.** The EQE for the waveguided plasmonic output in this case was calculated with the approach presented above.

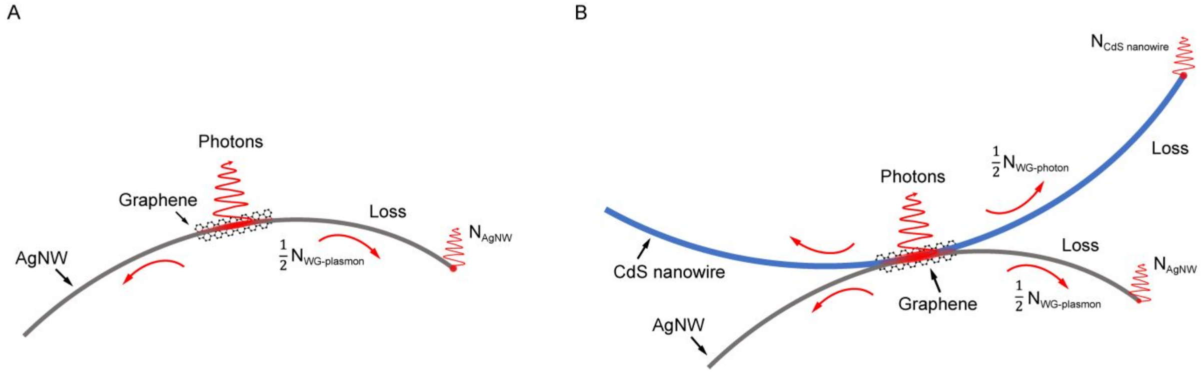

Figure S4. (**A**) Schematic diagram showing the outcoupling of IET-excited optical signals into the plasmonic AgNW waveguide, for the device presented in Figure 3. (**B**) Schematic diagram showing the outcoupling of IET-excited optical

signals into both the plasmonic AgNW and photonic CdS nanowire waveguides, for the device presented in Figure 4.

Section 7. Micromanipulation process of coupling of CdS nanowire with MIG-TJ

The micromanipulation process of coupling a CdS nanowire with a MIG-TJ was carried out under an optical microscope. A silica fiber taper was first fabricated by flame-assisted taper drawing of a standard optical fiber and mounted on a precisely controlled 3-dimensional translation stage. Then, a CdS nanowire with an appropriate diameter was picked up from a substrate and placed next to the MIG-TJ by the fiber taper. Subsequently, the CdS nanowire was gently pushed into contact with the tunnel junction region of the MIG-TJ. Finally, a laser light was coupled into one end of the AgNW to check the coupling between the AgNW and the CdS nanowire (light emission can be observed at the end of the CdS nanowire if they are coupled well).

Section 8. Setup for direct electrical modulation of light-emitting MIG-TJs

To demonstrate the possibility of direct electrical modulation of the optical output, as schematically shown in Figure S5, a bias with a square voltage waveform (switched between 0 and 2.5 V at a frequency of 1 kHz using a signal generator, Gw Instek GFG-8216A) was applied to a light-emitting MIG-TJ instead of a constant bias. The output light from a nanowire end was collected and directed through an optical fiber to a single photon counting module (SPCM) for detection.

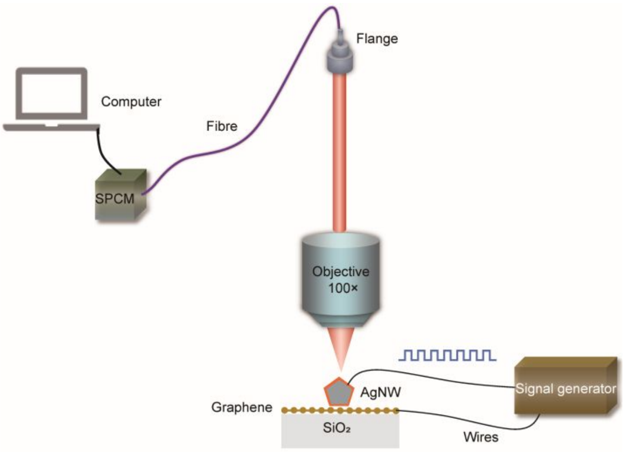

Figure S5. Schematic diagram of the setup for direct electrical modulation of a light-emitting MIG-TJ.

REFERENCES

1. Kern, J.; Kullock, R.; Prangsma, J.; Emmerling, M.; Kamp, M.; Hecht, B., Electrically driven optical antennas. *Nature Photon.* **2015,** *9* (9), 582-586.
2. Wang, P.; Krasavin, A. V.; Nasir, M. E.; Dickson, W.; Zayats, A. V., Reactive tunnel junctions in electrically driven plasmonic nanorod metamaterials. *Nature Nanotech.* **2018,** *13* (2), 159-164.
3. Parzefall, M.; Novotny, L., Light at the End of the Tunnel. *ACS Photonics* **2018,** *5* (11), 4195-4202.
4. Li, J.; Krasavin, A. V.; Webster, L.; Segovia, P.; Zayats, A. V.; Richards, D., Spectral variation of fluorescence lifetime near single metal nanoparticles. *Sci. Rep.* **2016,** *6* (1), 21349.
5. Johnson, P. B.; Christy, R. W., Optical Constants of the Noble Metals. *Phys. Rev. B* **1972,** *6* (12), 4370-4379.
6. Wang, Y.; Ma, Y.; Guo, X.; Tong, L., Single-mode plasmonic waveguiding properties of metal nanowires with dielectric substrates. *Opt. Express* **2012,** *20* (17), 19006-19015.
7. König, T. A. F.; Ledin, P. A.; Kerszulis, J.; Mahmoud, M. A.; El-Sayed, M. A.; Reynolds, J. R.; Tsukruk, V. V., Electrically Tunable Plasmonic Behavior of Nanocube–Polymer Nanomaterials Induced by a Redox-Active Electrochromic Polymer. *ACS Nano* **2014,** *8* (6), 6182-6192.
8. Emani, N. K.; Chung, T.-F.; Ni, X.; Kildishev, A. V.; Chen, Y. P.; Boltasseva, A., Electrically Tunable Damping of Plasmonic Resonances with Graphene. *Nano Lett.* **2012,** *12* (10), 5202-5206.
9. Falkovsky, L. A., Optical properties of graphene. *J. Phys. Conf. Ser.* **2008,** *129*, 012004.
10. Parzefall, M.; Szabó, Á.; Taniguchi, T.; Watanabe, K.; Luisier, M.; Novotny, L., Light from van der Waals quantum tunneling devices. *Nature Commun.* **2019,** *10* (1), 292.
11. Zomer, P. J.; Guimarães, M. H. D.; Brant, J. C.; Tombros, N.; van Wees, B. J., Fast pick up technique for high quality heterostructures of bilayer graphene and hexagonal boron nitride. *Appl. Phys. Lett.* **2014,** *105* (1), 013101.
12. Ferrari, A. C.; Meyer, J. C.; Scardaci, V.; Casiraghi, C.; Lazzeri, M.; Mauri, F.; Piscanec, S.; Jiang, D.; Novoselov, K. S.; Roth, S.; Geim, A. K., Raman Spectrum of Graphene and Graphene Layers. *Phys. Rev. Lett.* **2006,** *97* (18), 187401.
13. Wang, P.; Wang, Y.; Tong, L., Functionalized polymer nanofibers: a versatile platform for manipulating light at the nanoscale. *Light Sci. Appl.* **2013,** *2* (10), e102-e102.
